# Gastrointestinal Toxicities of Targeted Cancer Therapies: A Narrative Review

**DOI:** 10.7759/cureus.105842

**Published:** 2026-03-25

**Authors:** Eunice K Omeludike, Uchechukwu N Oguama, Nneoma Ubah, Cherechi O Nwabueze, Nzubechukwu Okeke, Eunice Aregbesola, Mesoma A Igbokwe, Meher Gujral, Ridwan A Lawal, Yetunde F Akande

**Affiliations:** 1 Internal Medicine, Piedmont Athens Regional Medical Center, Athens, USA; 2 Pharmacology, University of Portharcourt, Port Harcourt, NGA; 3 Internal Medicine, Montefiore St. Luke's Cornwall Hospital, Newburgh, USA; 4 Internal Medicine, John H. Stroger, Jr. Hospital of Cook County, Chicago, USA; 5 Public Health, George Washington University, Washington DC, USA; 6 Medical Education, University of Nigeria, Enugu, NGA; 7 Medicine, Nuvance Health, Poughkeepsie, USA; 8 General Medicine, University of Missouri, Columbia, USA; 9 Internal Medicine, Rochester General Hospital, Rochester, USA; 10 Internal Medicine, Zucker School of Medicine at Hofstra University/Northwell Health Vassar Brothers Medical Center, Poughkeepsie, USA; 11 Internal Medicine, University of Texas Rio Grande Valley School of Medicine, Edinburg, USA

**Keywords:** diarrhea, drug–drug interactions, gastrointestinal toxicity, pharmacist-led care, pharmacology, targeted cancer therapy

## Abstract

Targeted cancer therapies have transformed the management of solid and hematologic malignancies through pathway-specific disease control across diverse tumor types. Despite these advances, gastrointestinal toxicities remain among the most frequent and clinically consequential adverse effects associated with these agents, often leading to dose interruptions, treatment discontinuation, and reduced adherence. Diarrhea is the dose-limiting and most prevalent gastrointestinal adverse event among various classes of targeted therapies, and nausea, vomiting, oral mucositis, and less frequent high-impact events cause varying degrees of patient-related morbidity.

To address practical outpatient decision-making gaps, this narrative review synthesizes peer-reviewed literature identified primarily through PubMed/MEDLINE and supplemented by manual screening of reference lists from key guidelines, clinical trials, and expert reviews. This review proposes a pharmacology-informed Mechanism-Exposure-Severity-Escalation (MESE) framework to organize gastrointestinal toxicity assessment and management by linking drug class mechanisms and exposure-modifying factors to severity-based intervention and escalation thresholds. Evidence was based on clinical practice guidelines, pivotal randomized trials, supportive care intervention studies, quality of evidence on pharmacokinetic and drug-drug interaction studies, and on observational safety reports with a focus on pharmacist-relevant decisions in routine practice.

Across the literature, targeted therapy-associated diarrhea emerged as a mechanistically heterogeneous toxicity with substantial variability in incidence, severity, and management requirements across drug classes. Evidence supports early, structured antidiarrheal intervention, selected prophylactic strategies for predictable high-risk agents, and proactive identification of pharmacokinetic modifiers, including gastric acid suppression, that may influence drug exposure and clinical outcomes. Other gastrointestinal toxicities, including oral mucositis, nausea, vomiting, and rare high-impact complications, are integrated within the framework through guideline-directed supportive care, class-specific prevention strategies, and defined escalation pathways when clinically indicated. Collectively, these findings support a pharmacist-forward, pharmacology-informed approach to outpatient gastrointestinal toxicity management that integrates mechanism-aware assessment, exposure-risk evaluation, and clearly defined escalation thresholds to preserve treatment continuity while safeguarding patient safety.

## Introduction and background

Gastrointestinal toxicities are among the most frequently encountered adverse effects in patients receiving contemporary anticancer therapies and represent a major determinant of treatment tolerability in routine clinical practice [1]. Clinical guidance consistently identifies diarrhea as a leading contributor to treatment-related morbidity in cancer populations, with the potential to cause dehydration, electrolyte imbalance, hospitalization, and premature interruption of anticancer therapy when not managed appropriately [2]. Accordingly, structured assessment and early supportive care are emphasized as essential components of toxicity management across oncology settings [2,3].

Although diarrhea is often discussed as a single clinical entity, its presentation and clinical significance in cancer patients are heterogeneous. Existing guidelines highlight that symptom severity, duration, and risk vary substantially according to treatment type, patient-related factors, and concurrent therapies, necessitating individualized assessment rather than uniform management approaches [2,3]. Other gastrointestinal toxicities, including nausea, vomiting, and oral mucositis, frequently coexist and may further compromise oral intake, functional status, and quality of life, but differ in their predictability, escalation thresholds, and implications for treatment continuity [1,3].

Within this broader gastrointestinal toxicity landscape, targeted cancer therapies warrant focused consideration. Reviews of targeted therapy-associated gastrointestinal adverse events consistently describe diarrhea as a common and often dose-limiting toxicity with clinically meaningful consequences for adherence and dose intensity across multiple drug classes [4]. Importantly, toxicity patterns differ substantially between agents, reinforcing the need for proactive, treatment-specific management strategies rather than generalized supportive care alone [4,5]. Comparative analyses further demonstrate that diarrhea associated with small-molecule tyrosine kinase inhibitors differs in frequency and clinical behavior from that observed with conventional cytotoxic chemotherapy, underscoring the relevance of drug-specific and pharmacologic factors in toxicity assessment [6].

Mechanistic investigations indicate that targeted therapy-associated diarrhea often reflects disruption of epithelial signaling pathways and alterations in intestinal secretory and absorptive processes rather than nonspecific mucosal injury alone [4,7]. These pathway-specific effects help explain observed variability in toxicity onset, severity, and persistence across drug classes and provide a rationale for tailoring management strategies to the pharmacologic properties of individual agents.

Accurate attribution of gastrointestinal symptoms therefore requires careful differential diagnosis. While treatment-related mechanisms account for many cases of diarrhea, clinical guidance emphasizes that alternative etiologies must be considered, particularly in patients presenting with severe, persistent, or atypical symptoms [3]. Infectious diarrhea remains an important diagnostic consideration in cancer patients, and appropriate evaluation is recommended when clinical features suggest an infectious or inflammatory process rather than isolated drug toxicity [8].

These distinctions have direct implications for outpatient management. Effective mitigation of gastrointestinal toxicities requires integration of class-specific risk patterns, anticipated timing of symptom onset, and pharmacologic modifiers that influence drug exposure and toxicity [4,5]. In particular, exposure-modifying factors such as drug-drug interactions and concomitant medications may meaningfully alter both efficacy and tolerability, yet are not consistently incorporated into routine toxicity assessment.

Pharmacists play a central role in translating pharmacologic understanding into practical outpatient toxicity management. Key activities include medication reconciliation, identification of drug-drug interactions and pharmacokinetic risk factors, toxicity triage during outpatient encounters, patient counseling on early symptom recognition, and implementation of guideline-informed supportive care strategies [2,3]. Within the framework proposed in this review, these activities operationalize pharmacology-informed toxicity assessment and support timely intervention when symptoms emerge. In targeted therapy settings, attention to pharmacologic and pharmacokinetic considerations helps inform toxicity attribution, monitoring intensity, and escalation decisions when routine measures are insufficient [4,5].

Accordingly, this narrative review synthesizes current evidence on gastrointestinal toxicities associated with targeted cancer therapies, with a primary focus on diarrhea as the most prevalent and clinically consequential adverse event. Unlike prior reviews that primarily catalog gastrointestinal adverse events by drug class, this review integrates pharmacologic mechanism, exposure-modifying factors, and escalation thresholds into a unified outpatient decision-making framework. Although the primary emphasis is on targeted therapy-associated toxicities, immune checkpoint inhibitor-related gastrointestinal toxicities are also briefly discussed later to support differential diagnosis and outpatient triage when patients receive multiple systemic therapies. The review is organized around a pharmacology-informed Mechanism-Exposure-Severity-Escalation (MESE) framework that links drug class and pharmacologic properties to monitoring intensity, selective prophylaxis for high-risk agents, and escalation thresholds for persistent, atypical, or severe symptoms. This framework is intended to support structured clinical reasoning rather than function as a prescriptive algorithm.

Table 1 summarizes the MESE framework for outpatient gastrointestinal toxicity management in targeted cancer therapy, outlining key pharmacologic and clinical inputs and their corresponding implications for monitoring, supportive care, and escalation decisions in routine practice.

**Table 1:** Mechanism–Exposure–Severity–Escalation (MESE) framework for outpatient gastrointestinal toxicity management in targeted cancer therapy The MESE framework is intended to support structured clinical reasoning and outpatient decision-making rather than function as a prescriptive algorithm. In practice, mechanism and exposure inform toxicity interpretation, while severity and escalation guide intervention and referral decisions. Credits: Created by the authors (informed by narrative review reporting guidance) [9-12].

Framework component	Key considerations (inputs)	Clinical implications (outputs)
Mechanism	Drug class and mechanism of action (e.g., pathway inhibition, epithelial signaling effects)	Anticipated GI toxicity pattern; selection of mechanism-appropriate supportive care
Exposure	Pharmacokinetic modifiers (e.g., gastric acid suppression, drug–drug interactions)	Adjustment of monitoring intensity; medication reconciliation and counseling priorities
Severity	Symptom grade, persistence, functional impact	Timing and intensity of intervention; reassessment versus continued outpatient management
Escalation	Atypical features, refractoriness, red-flag symptoms	Treatment interruption, diagnostic evaluation, or urgent referral

## Review

Study design and reporting approach

This article was developed as a narrative review synthesizing clinically relevant evidence on gastrointestinal toxicities associated with targeted cancer therapies, with emphasis on pharmacologic mechanisms and practical outpatient management considerations [9]. The approach was guided by established recommendations for narrative biomedical reviews, prioritizing transparency of sources while avoiding systematic-review style claims [10]. A clear narrative structure was maintained to emphasize clinical applicability and drug-specific management implications relevant to pharmacy practice [11]. Reporting choices were additionally informed by SANRA principles to support clarity, appropriate referencing, and balanced synthesis, without implying formal scoring, quantitative appraisal, or exhaustive coverage [12].

Literature sources and search strategy

Searches were performed in PubMed/MEDLINE from database inception through January 18, 2026, which served as the primary database because of its comprehensive indexing of biomedical and oncology literature. Search terms focused on gastrointestinal toxicities associated with targeted cancer therapies, including diarrhea, mucositis, nausea, vomiting, colitis, and related management strategies, combined with terms describing targeted systemic anticancer agents such as tyrosine kinase inhibitors, pathway-specific inhibitors, monoclonal antibodies, and antibody-drug conjugates.

An example PubMed query was: ("targeted therapy" OR tyrosine kinase inhibitor OR monoclonal antibody OR "antibody-drug conjugate" OR pathway inhibitor) AND (diarrhea OR diarrhoea OR gastrointestinal toxicity OR mucositis OR stomatitis OR nausea OR vomiting OR colitis OR perforation) AND (management OR supportive care OR prophylaxis OR pharmacologic OR pharmacokinetic OR interaction)

Reference lists of relevant clinical practice guidelines, reviews, and key clinical studies were manually screened to identify additional pertinent literature. The review prioritized peer-reviewed clinical studies and guideline documents relevant to gastrointestinal toxicity mechanisms and outpatient management considerations. Grey literature and conference abstracts were not systematically included because the synthesis focused on established peer-reviewed evidence.

Study selection and evidence synthesis

Articles were prioritized if they addressed gastrointestinal adverse events or management strategies in adult cancer populations receiving targeted systemic therapies. Priority was given to clinical practice guidelines, randomized trials, and supportive care intervention studies where available, followed by pharmacologic and mechanistic investigations, pharmacokinetic and drug-drug interaction analyses, and observational safety reports that informed outpatient management considerations. Literature identification and interpretation were conducted collaboratively by the authors, with prioritization guided by relevance to gastrointestinal toxicity mechanisms, outpatient management implications, and pharmacist-relevant clinical decision points such as early intervention, interaction risk identification, and escalation thresholds.

Selected agents and clinical contexts were discussed in greater depth as illustrative exemplars where interventional evidence, well-characterized toxicity patterns, or pharmacokinetic considerations provided clearer outpatient management guidance. These examples were selected to illustrate clinically relevant mechanisms and management principles rather than to provide exhaustive coverage of all targeted therapies. During synthesis, evidence strength was considered qualitatively, with greater emphasis placed on clinical practice guidelines, prospective trials, and supportive care intervention studies where available.

When evidence was heterogeneous or conflicting, findings were interpreted cautiously and contextualized according to study design, population, and strength of inference. Evidence was synthesized narratively to support the MESE framework, linking pharmacologic mechanism and exposure modifiers to severity assessment and escalation decisions. Formal meta-analysis, pooled estimates, and structured risk-of-bias assessment were not performed, and conclusions were framed conservatively when data were indirect or evolving.

To enhance interpretive clarity, evidence was weighted by recency and clinical relevance. More recent publications were prioritized when reflecting current practice, while foundational studies were retained where they provided enduring mechanistic insights. The review was limited to English-language studies in adult cancer populations; pediatric studies were excluded due to substantially different toxicity profiles and management pathways.

Evidence Map

The evidence informing gastrointestinal toxicity management in targeted cancer therapy is derived from complementary sources that differ in evidentiary strength and clinical applicability. This evidence map represents a conceptual classification of the types of sources informing the narrative synthesis rather than a structured mapping of all identified studies. Supportive care reviews characterize class-associated gastrointestinal toxicity patterns and outline general outpatient mitigation strategies, consistently identifying diarrhea as a clinically disruptive signal [13]. Guideline-based evidence provides structured recommendations for gastrointestinal toxicities that commonly coexist with diarrhea, including oral mucositis and stomatitis, where consensus-driven prevention and treatment strategies are broadly applicable across oncology settings [14].

Higher-level interventional evidence is available for selected, predictable toxicity syndromes, such as prophylactic strategies for mTOR inhibitor-associated stomatitis evaluated in prospective clinical studies [15]. Antiemetic guideline frameworks, although originally developed for cytotoxic chemotherapy, remain relevant in targeted therapy settings where nausea and vomiting occur and may compound diarrhea-related morbidity, supporting risk-adapted rather than uniform supportive care approaches [16-20]. Mechanistic and pharmacologic literature further contextualizes agent-specific variability in toxicity profiles and supports individualized, class-aware management rather than symptom-based escalation alone [13].

Mechanistic Determinants Informing Gastrointestinal Toxicity Assessment

Gastrointestinal toxicities associated with targeted cancer therapies arise from pharmacologic mechanisms that differ fundamentally from the nonspecific mucosal injury typical of cytotoxic chemotherapy. Reviews emphasize that diarrhea and related gastrointestinal symptoms often reflect pathway-specific effects on intestinal epithelial function rather than epithelial cell death alone [4]. These distinctions explain the variability in toxicity onset, severity, and clinical course observed across targeted agents and drug classes.

Disruption of growth factor signaling pathways plays a central role in diarrhea associated with several targeted therapies. Inhibition of epidermal growth factor receptor family signaling alters epithelial turnover, barrier integrity, and ion transport, leading to changes in secretion and absorption within the gastrointestinal tract [4,7]. Clinically, this manifests as a predominantly secretory diarrhea phenotype that may persist despite dietary modification and often requires early pharmacologic intervention.

Targeted therapies may also influence intestinal physiology through additional mechanisms, including altered epithelial ion transport processes such as chloride secretion, water transport, and epithelial tight junction function. Experimental and translational studies support a mechanistic basis for diarrhea distinct from inflammatory or infectious processes [7]. As a result, antidiarrheal strategies effective for chemotherapy-associated mucosal injury may be insufficient when symptoms reflect ongoing secretory or transport-mediated dysfunction.

Host-related and treatment-related factors further modulate susceptibility to gastrointestinal toxicity. Comparative analyses demonstrate that diarrhea associated with small-molecule tyrosine kinase inhibitors differs in pattern and frequency from that observed with cytotoxic chemotherapy, indicating interactions between drug-specific properties and patient-level factors [6]. Although emerging research has explored potential contributions of the intestinal microbiome to diarrhea susceptibility, available data remain exploratory and should not inform routine clinical decision-making at this time [6].

Integrating mechanistic understanding into clinical assessment supports more nuanced management decisions within the MESE framework. Diarrhea consistent with expected, mechanism-linked toxicity may be managed with structured supportive care and close monitoring, whereas severe, atypical, progressive, or refractory symptoms should prompt reassessment for alternative etiologies or treatment-related complications [3]. In practical terms, mechanistic distinctions inform the “mechanism” component of the framework, guiding early supportive care selection, monitoring intensity, and escalation thresholds when symptoms fail to respond to routine outpatient management.

Diarrhea as the Dominant Gastrointestinal Toxicity: Clinical Patterns and Grading

This review prioritizes diarrhea as the dominant gastrointestinal toxicity associated with targeted cancer therapies because it is the most frequent, treatment-limiting, and operationally consequential adverse event encountered in outpatient practice. Across multiple targeted therapy classes, diarrhea accounts for a substantial proportion of dose interruptions, reductions, and unplanned healthcare utilization, and often represents the earliest toxicity signal requiring active pharmacologic intervention. Other gastrointestinal toxicities, including oral mucositis, immune-mediated colitis, and gastrointestinal perforation, are clinically important but are either more class-restricted, less prevalent, or managed through distinct diagnostic and escalation pathways. Emphasis on diarrhea therefore reflects its disproportionate impact on treatment continuity, patient burden, and pharmacist-led outpatient toxicity management.

Across targeted cancer therapy classes, diarrhea consistently emerges as the most prevalent and clinically disruptive gastrointestinal adverse event. Clinical practice guidelines and observational data identify diarrhea as a frequent driver of dose interruption, dose reduction, or early discontinuation when symptoms are not anticipated or managed promptly [17]. This burden is particularly pronounced in outpatient settings, where delayed symptom reporting and underestimation of severity can allow progression before timely intervention.

The clinical severity of targeted therapy-associated diarrhea varies widely and is commonly graded using standardized toxicity criteria, such as the Common Terminology Criteria for Adverse Events (CTCAE), to guide management decisions. Guideline frameworks emphasize distinguishing mild, self-limited symptoms from persistent or higher-grade diarrhea associated with dehydration, electrolyte abnormalities, or functional decline, as these features directly inform escalation of care and treatment modification [17,18]. Early recognition of worsening severity is therefore central to preventing avoidable hospitalization and disruption of anticancer therapy.

Importantly, diarrhea associated with targeted therapies differs in clinical behavior from that observed with cytotoxic chemotherapy. Some agents produce early-onset diarrhea that may stabilize with continued therapy and appropriate supportive care, whereas others are associated with delayed or cumulative toxicity requiring sustained monitoring and repeated intervention [4,6]. These differences underscore the need for agent- and class-aware assessment rather than uniform symptom management across regimens.

Guideline-based management recommendations emphasize early initiation of antidiarrheal therapy, adequate hydration, and close outpatient monitoring, with escalation recommended when symptoms persist despite first-line measures or when red-flag features emerge [17,18]. In such cases, treatment interruption and further diagnostic evaluation may be warranted to exclude alternative etiologies or clinically significant treatment-related injury.

Although diarrhea is common in this population, not all cases are attributable solely to targeted therapy-related mechanisms. Clinical guidance stresses reassessment of symptom etiology when diarrhea is severe, prolonged, progressive, or atypical, particularly in patients receiving multiple systemic therapies or concomitant medications with gastrointestinal effects [17]. Ongoing reassessment, rather than reflexive attribution to anticancer treatment alone, is therefore essential to safe and effective outpatient toxicity management.

Pharmacologic Management of Targeted Therapy-Associated Diarrhea

Pharmacologic management of diarrhea in patients receiving targeted cancer therapies is guided primarily by symptom severity, persistence, and the presence of complications rather than a single uniform algorithm. Although many supportive care guideline frameworks were initially developed for chemotherapy-associated gastrointestinal toxicities, they provide transferable principles for outpatient assessment, stepwise pharmacologic intervention, and escalation thresholds that are applicable to targeted therapy-associated diarrhea.

Clinical practice guidelines emphasize early initiation of antidiarrheal therapy alongside supportive measures such as oral hydration and electrolyte replacement to prevent progression to more severe diarrhea requiring hospitalization or treatment interruption [17,18]. In practice, management generally follows a stepwise approach in which initial antidiarrheal therapy and supportive care represent first-line treatment, with escalation measures or treatment interruption considered when symptoms persist or progress despite these measures. Ongoing clinical monitoring is essential, as symptom trajectory over time often provides more actionable information than a single assessment.

Treatment interruption or dose modification is recommended when diarrhea reaches moderate to severe grades or fails to respond to appropriate supportive care. Guidance documents caution that continuation of therapy in the setting of uncontrolled diarrhea increases the risk of dehydration, renal dysfunction, and cumulative toxicity, particularly during prolonged outpatient treatment [17,18]. Pharmacologic management should therefore be integrated with treatment-holding criteria and structured reassessment rather than pursued in isolation.

Mechanistic considerations further inform decision-making. Differences in onset, severity, and duration of diarrhea across targeted therapy classes influence both expected response to antidiarrheal therapy and escalation needs [4,6]. Diarrhea consistent with anticipated, mechanism-linked toxicity may be managed with structured supportive care and close follow-up, whereas progressive, refractory, or discordant symptoms should prompt earlier reassessment rather than repeated escalation of antidiarrheal therapy alone.

Reevaluation of symptom etiology is essential when diarrhea is severe, atypical, or refractory to standard pharmacologic management. Clinical guidance emphasizes consideration of alternative causes, including infection, concomitant medications, or overlapping treatment-related toxicities, before further intensification of antidiarrheal therapy or continuation of the targeted agent [17]. This approach reduces inappropriate attribution of symptoms to targeted therapy alone and supports safer, more effective outpatient management.

Drug-Specific Prophylaxis and High-Risk Settings

While general supportive care principles form the foundation of diarrhea management, certain targeted cancer therapies are associated with predictable gastrointestinal toxicity profiles that warrant proactive, drug-specific strategies. These examples also illustrate how different components of the MESE framework, including mechanism-linked toxicity patterns, exposure-related risk factors, severity assessment, and escalation thresholds, inform outpatient management decisions in specific clinical contexts. The strength of evidence supporting these approaches varies across examples, including prospective or interventional studies, clinical practice guideline recommendations, and broader extrapolation of supportive care principles. Supportive care guidance emphasizes that a purely reactive approach may be insufficient in patients at high risk of treatment-related diarrhea, particularly when toxicity interferes with adherence or dose intensity [13]. Contemporary gastrointestinal supportive care frameworks further recognize that guideline-consistent prophylaxis and early intervention can reduce symptom burden and improve tolerability in selected high-risk settings, rather than being applied uniformly across all regimens [19,20].

Human Epidermal Growth Factor Receptor 2 (HER2)-Directed Therapy: Neratinib-Associated Diarrhea

Neratinib is consistently associated with a high incidence of early-onset diarrhea, particularly during initial treatment cycles. In the ExteNET trial, diarrhea was reported as a frequent adverse event with clinically meaningful impact on treatment tolerability, establishing this toxicity as predictable and clinically relevant at therapy initiation [21]. These findings underscore the need for anticipatory rather than reactive management when initiating neratinib.

Subsequent prospective studies demonstrated that structured prophylactic approaches can substantially mitigate this risk. The CONTROL trial evaluated multiple protocolized prophylactic regimens and showed reductions in diarrhea incidence and severity compared with historical experience, alongside improved treatment continuation [22]. Updated analyses further supported the benefit of tailored prophylactic strategies across intervention arms [23]. More recent supportive care studies have explored adjunctive agents such as crofelemer, expanding evidence-based options for diarrhea management in selected patients receiving neratinib [24]. Collectively, these data support proactive prophylaxis as a standard component of neratinib initiation rather than delayed escalation after symptom onset.

More broadly, these findings highlight that tolerability considerations are integral to maintaining extended or intensified targeted therapy regimens and should be considered alongside efficacy when selecting and sustaining treatment [25].

Mechanistic Target of Rapamycin (mTOR) Inhibition and Oral Mucosal Toxicity

Although diarrhea may occur with mTOR inhibitors, oral mucositis and stomatitis represent the most prominent gastrointestinal toxicities in this class. Prospective evaluation of dexamethasone mouthwash demonstrated a reduction in the incidence and severity of everolimus-associated stomatitis, supporting a preventive rather than reactive management approach at treatment initiation [15]. This example illustrates how identification of class-specific toxicity patterns enables targeted prophylaxis where interventional evidence exists, without extending prophylactic strategies indiscriminately across therapies.

Cyclin-Dependent Kinase 4/6 (CDK4/6) Inhibitors and Agent-Specific Risk

Within the CDK4/6 inhibitor class, gastrointestinal toxicity risk is not uniform. Safety analyses from randomized clinical trials indicate that abemaciclib is associated with a higher incidence of diarrhea compared with other CDK4/6 inhibitors, necessitating closer monitoring and earlier supportive care intervention [26]. Recognition of this agent-specific risk allows clinicians and pharmacists to individualize counseling, monitoring intensity, and early management strategies rather than relying on class-wide assumptions.

Other Targeted Classes for Class Mapping and GI Risk

Beyond agents with well-defined prophylactic strategies, several targeted therapy classes illustrate how gastrointestinal risk varies in mechanism, severity, and escalation requirements. These examples are included to support class-aware risk mapping rather than to provide comprehensive toxicity profiles.

Phosphatidylinositol 3-Kinase (PI3K) Inhibition: Alpelisib

Targeted PI3K pathway inhibition can produce gastrointestinal toxicities that interact with other clinically meaningful adverse effects, creating compounding outpatient risk. In the pivotal study of alpelisib in PIK3CA-mutated, hormone receptor-positive advanced breast cancer, gastrointestinal adverse events formed part of a broader safety profile necessitating early supportive care planning and structured monitoring at therapy initiation [27]. From a pharmacist perspective, this reinforces the importance of proactive counseling, early symptom reporting, and coordinated follow-up when multiple adverse effects may converge to increase dehydration or treatment interruption risk.

Phosphatidylinositol 3-Kinase Delta Inhibition: Idelalisib

Certain targeted agents are characterized less by frequent low-grade toxicity and more by clearly defined escalation thresholds. Expert guidance on idelalisib-associated adverse events emphasizes structured monitoring and management pathways, reflecting the importance of recognizing when toxicity patterns warrant treatment interruption and higher-level evaluation rather than prolonged outpatient symptom suppression [28]. In this context, persistent or worsening diarrhea may signal clinically significant toxicity requiring escalation.

Anti-vascular Endothelial Growth Factor (VEGF) Therapy: Gastrointestinal Perforation as a High-Impact Event

Although less common than diarrhea, gastrointestinal perforation represents a rare but serious adverse event associated with anti-VEGF therapy. A meta-analysis evaluating bevacizumab-associated gastrointestinal perforation characterized this risk across cancer populations and underscored the need for heightened clinical vigilance when patients present with severe or atypical abdominal symptoms [29]. Clinical commentary further emphasizes that features such as acute abdominal pain, peritonitis, or rapid clinical deterioration should prompt immediate evaluation and escalation rather than routine outpatient management [30].

Tyrosine Kinase Inhibitors in Hepatocellular Carcinoma

Targeted therapies used in hepatocellular carcinoma are frequently administered over extended durations, making tolerability management a key determinant of treatment persistence. Reviews of tyrosine kinase inhibitor-associated adverse events in this setting emphasize structured toxicity mitigation strategies and reinforce that effective adverse event management is integral to maintaining therapy in real-world practice [31]. This supports pharmacist-led monitoring approaches that integrate gastrointestinal symptom assessment with medication reconciliation and supportive care over prolonged treatment courses.

Antibody-Drug Conjugates and Gastrointestinal Adverse Events

As antibody-drug conjugates are increasingly incorporated into treatment paradigms across multiple malignancies, pooled analyses highlight gastrointestinal toxicity as part of their broader adverse event landscape [32]. These data support anticipation of GI adverse events and management within class-aware supportive care frameworks, rather than treating such toxicities as unexpected. In practice, this reinforces early counseling, symptom monitoring, and timely escalation when toxicity becomes persistent or clinically significant.

Drug-Drug Interactions and Pharmacokinetic Modifiers in Gastrointestinal Toxicity Management

Pharmacokinetic interactions represent an important and often underrecognized contributor to gastrointestinal toxicity risk and treatment failure in patients receiving oral targeted cancer therapies. Several tyrosine kinase inhibitors exhibit pH-dependent absorption, rendering systemic drug exposure vulnerable to concomitant gastric acid-suppressive therapy [33]. In this context, altered absorption may influence not only therapeutic efficacy but also toxicity patterns and symptom persistence. Beyond pH-dependent absorption, other pharmacokinetic modifiers, including CYP-mediated interactions and transporter-related effects, may further influence drug exposure and clinical outcomes.

Clinical pharmacokinetic studies demonstrate that proton pump inhibitor coadministration can significantly reduce erlotinib exposure, raising concerns regarding diminished efficacy and altered clinical outcomes when drug absorption is impaired [33]. Observational analyses further associate gastric acid suppression with reduced clinical effectiveness of erlotinib in non-small-cell lung cancer, reinforcing the real-world relevance of this interaction beyond controlled study settings [34]. These findings underscore the importance of interpreting gastrointestinal symptoms in the broader context of drug exposure rather than attributing toxicity solely to the targeted agent itself.

Interventional studies have explored mitigation strategies, including the use of acidic beverages to transiently enhance erlotinib absorption in patients receiving acid-suppressive therapy. Although partial restoration of drug exposure has been demonstrated under controlled conditions, these approaches require careful patient selection and counseling and should not be applied indiscriminately in routine practice [35]. Additional pharmacokinetic analyses confirm that proton pump inhibitors lower erlotinib plasma concentrations, supporting proactive medication review when initiating or continuing therapy [36].

Expert pharmacologic commentary emphasizes that gastric acid-suppressive therapy should be reassessed for clinical necessity in patients receiving pH-sensitive targeted agents rather than continued by default [37]. Case-based discussions further illustrate that nonprescription medications and dietary factors may meaningfully influence drug absorption, underscoring the importance of structured counseling, medication reconciliation, and ongoing monitoring in outpatient oncology care [38,39].

High-Impact Gastrointestinal Events and Escalation Thresholds 

While diarrhea represents the most frequent gastrointestinal toxicity associated with targeted cancer therapies, clinicians must remain vigilant for less common but potentially life-threatening gastrointestinal adverse events that require immediate escalation rather than outpatient symptom control. Anti-VEGF therapies, particularly bevacizumab, have been associated with an increased risk of gastrointestinal perforation across cancer populations [29].

Meta-analytic evidence characterizes gastrointestinal perforation as a rare but serious complication with substantial morbidity and mortality, underscoring the importance of early recognition and prompt intervention [29]. Risk may be influenced by underlying tumor involvement of the gastrointestinal tract, prior abdominal interventions, or concurrent treatments, although predictive factors remain variably defined across studies. Clinical commentary further emphasizes that severe or localized abdominal pain, signs of peritonitis, gastrointestinal bleeding, or rapid clinical deterioration in patients receiving anti-VEGF therapy should prompt urgent evaluation rather than continuation of supportive care measures alone [30].

From a pharmacologic and clinical workflow perspective, these events delineate the limits of routine antidiarrheal management. Gastrointestinal symptoms that are severe, atypical, or discordant with expected toxicity patterns, particularly in high-risk treatment contexts, should trigger reassessment for alternative or emergent diagnoses such as perforation, ischemia, or inflammatory complications. Pharmacists play a critical role in this safety framework by identifying red-flag features during outpatient encounters, reinforcing escalation thresholds, and facilitating timely referral when supportive care is no longer appropriate.

Diagnostic Distinction: Immune-Mediated Gastrointestinal Toxicity

Although immune checkpoint inhibitors are not classified as targeted cancer therapies, they are increasingly encountered alongside targeted agents in contemporary oncology practice and are associated with distinct gastrointestinal toxicities that require fundamentally different management strategies. Immune-mediated colitis represents a clinically significant adverse event driven by inflammatory mechanisms, in contrast to the predominantly secretory or exposure-related diarrhea observed with kinase inhibition [40].

Clinical practice updates emphasize that immune-mediated colitis should be suspected in patients receiving checkpoint inhibitors who develop persistent or progressive diarrhea, abdominal pain, or gastrointestinal bleeding, particularly when symptoms are refractory to standard antidiarrheal therapy [40]. In these settings, escalation to diagnostic evaluation and immunosuppressive management is recommended rather than continued outpatient supportive care alone. Depending on the clinical presentation, evaluation may include stool studies, imaging, or endoscopic assessment to distinguish immune-mediated toxicity from alternative etiologies.

American Society of Clinical Oncology (ASCO) clinical practice guidelines further reinforce that immune-related gastrointestinal adverse events require early recognition, severity grading, and prompt initiation of corticosteroid therapy in moderate to severe cases to prevent complications such as perforation, prolonged morbidity, or treatment-related mortality [41]. Updated guidance highlights refinements in management pathways, including criteria for treatment interruption, steroid tapering, and specialist referral [42].

This distinction is operationally important for pharmacists involved in outpatient toxicity triage. Failure to differentiate immune-mediated colitis from targeted therapy-associated diarrhea may delay appropriate escalation and expose patients to unnecessary risk. Accordingly, immune checkpoint inhibitor-related gastrointestinal toxicity is included here as a diagnostic safeguard to support safe triage and escalation while remaining conceptually distinct from targeted therapy-associated gastrointestinal adverse events.

Clinical Context: Prolonged Targeted Therapy Exposure and Toxicity Relevance 

The clinical relevance of gastrointestinal toxicity management must be interpreted in the context of prolonged exposure to modern targeted cancer therapies, many of which are administered continuously in the outpatient setting. Randomized trials of first-line epidermal growth factor receptor-targeted therapies in advanced non-small-cell lung cancer demonstrate meaningful survival improvements that translate into longer treatment durations and sustained exposure to therapy-related adverse events [43,44].

Comparative studies and accompanying commentaries highlight that extended treatment exposure amplifies the clinical importance of cumulative and chronic toxicities, including gastrointestinal adverse events that may be under-recognized in short-term efficacy-focused assessments [45-47]. In this setting, tolerability becomes a determinant of treatment persistence and real-world benefit, underscoring that proactive gastrointestinal toxicity management is essential to maintaining therapeutic intensity and functional status over time [48,49]. In routine clinical practice, treatment adherence and dose modification decisions are closely linked to toxicity burden, reinforcing the importance of early symptom control and structured outpatient management.

Table 2 summarizes class-specific gastrointestinal toxicity patterns associated with commonly used targeted cancer therapies, highlighting dominant toxicity profiles, key risk characteristics, and corresponding management priorities relevant to outpatient practice.

**Table 2:** Class-specific gastrointestinal toxicity patterns and management priorities in targeted cancer therapy Management emphases reflect varying levels of evidence, including guideline-based, prospective, observational, and expert-informed sources. EGFR, epidermal growth factor receptor; HER2, human epidermal growth factor receptor 2; TKI, tyrosine kinase inhibitor; mTOR, mechanistic target of rapamycin; PI3K, phosphatidylinositol 3-kinase; VEGF, vascular endothelial growth factor. Credits: Created by the authors based on the cited sources [4,5,15,21-23,26-29,32-34,40-44].

Targeted therapy class/agent (illustrative)	Dominant GI toxicity pattern	Key risk characteristics	Management emphasis within the MESE framework	Key references
EGFR/HER family TKIs (e.g., erlotinib)	Secretory diarrhea	pH-dependent absorption; exposure variability with acid suppression	Exposure review (acid suppression), early antidiarrheal therapy, close outpatient monitoring	[4,5,33,34]
HER2-directed TKI (neratinib)	Early-onset, high-frequency diarrhea	Predictable early toxicity; dose-limiting without prophylaxis	Prophylaxis at initiation, early monitoring, escalation if refractory	[21–23]
CDK4/6 inhibitors (abemaciclib)	Dose-related diarrhea	Agent-specific risk within class	Early counseling, low threshold for intervention, ongoing reassessment	[26]
mTOR inhibitors (everolimus)	Oral mucositis / stomatitis > diarrhea	Predictable mucosal toxicity	Preventive strategy at initiation (e.g., topical prophylaxis)	[15]
PI3K inhibitors (alpelisib)	Diarrhea with metabolic comorbidity	Compounding outpatient risk (dehydration, treatment interruption)	Early supportive care, structured follow-up, escalation if persistent	[27]
PI3Kδ inhibitors (idelalisib)	Severe or inflammatory diarrhea	May signal clinically significant toxicity	Early escalation, treatment interruption, diagnostic evaluation	[28]
Anti-VEGF therapy (bevacizumab)	GI perforation (rare but high-impact)	Life-threatening complication	Immediate escalation, avoid outpatient symptom suppression	[29,30]
Antibody–drug conjugates (class effect)	Variable GI toxicity	Increasing use; heterogeneous risk	Anticipatory counseling, symptom monitoring, timely escalation	[32]

Discussion

This narrative review synthesizes evidence on gastrointestinal toxicities associated with targeted cancer therapies and emphasizes practical, pharmacology-informed strategies that support treatment continuity in outpatient care. Across guideline frameworks and clinical literature, diarrhea consistently emerges as the most clinically consequential gastrointestinal adverse event. When symptoms are not anticipated or addressed early, diarrhea has direct implications for adherence, dose intensity, and healthcare utilization [1,2].

A central insight of this review is that gastrointestinal toxicities associated with targeted therapies are not uniform across drug classes. In this context, the MESE framework extends existing supportive care approaches by explicitly integrating pharmacologic mechanism, exposure-related modifiers, and severity-based escalation into a unified structure for outpatient clinical decision-making. Effective management must therefore reflect mechanistic and agent-specific differences rather than rely on a single generic supportive care approach. Variability in onset, severity, and clinical course across agents highlights the importance of mechanism-aware assessment. Symptoms that persist despite appropriate initial measures should prompt reassessment rather than prolonged outpatient symptom suppression [4,7].

Unlike prior reviews that primarily catalog gastrointestinal adverse events by drug class, this review integrates pharmacologic mechanism, exposure-modifying factors, and escalation thresholds into a unified outpatient decision-making framework. By explicitly incorporating pharmacokinetic modifiers and drug-drug interactions into toxicity assessment, the MESE framework highlights exposure-related contributors to both efficacy and tolerability that may otherwise be overlooked in routine practice.

Evidence from the erlotinib-acid suppression literature illustrates that concomitant gastric acid-reducing therapy can meaningfully reduce exposure to certain orally administered targeted agents, with implications for both treatment effectiveness and toxicity persistence [33,34]. These findings underscore the importance of comprehensive medication reconciliation, including nonprescription agents, as a routine component of outpatient oncology care.

High-risk clinical settings in which gastrointestinal toxicity is predictable and supported by interventional evidence represent another area where a pharmacist-forward approach adds value. Neratinib-associated diarrhea exemplifies a setting in which protocolized prophylaxis improves tolerability and reduces early treatment disruption, supporting a preventive rather than reactive management model for selected high-risk agents [22,23].

Although this review focuses on targeted cancer therapies, outpatient toxicity triage must also account for immune checkpoint inhibitor-related gastrointestinal toxicity, which is mechanistically distinct and requires different escalation pathways. Recognition of immune-mediated colitis is essential to prevent misclassification and delayed escalation, particularly in patients receiving multiple systemic therapies over time [40,41].

As targeted therapies are administered for longer durations and across broader patient populations, tolerability management assumes increasing importance. Survival gains associated with modern first-line targeted therapies translate into prolonged exposure to therapy-related adverse events, underscoring the need for proactive gastrointestinal toxicity management to maintain therapeutic intensity and real-world benefit [43,47].

From a pharmacist-forward perspective, the evidence summarized here supports a structured outpatient workflow that integrates two complementary elements: pharmacologic understanding of mechanism-based toxicity patterns and practice-level pharmacist interventions that guide monitoring, medication review, supportive care implementation, and escalation decisions. Viewed through the MESE framework, pharmacologic mechanism and exposure modifiers inform interpretation of toxicity patterns and monitoring priorities, while severity assessment guides the timing and intensity of supportive care intervention. Escalation criteria help distinguish routine toxicity from high-risk presentations requiring treatment interruption, diagnostic evaluation, or urgent referral. Operational priorities therefore include early symptom recognition and grading, prompt initiation of evidence-based supportive care, medication reconciliation focused on interaction risks and contributory agents, selective use of drug-specific prophylaxis in high-risk settings, and timely referral when symptoms are atypical, progressive, or refractory to initial management. In practice, these activities are operationalized through pharmacist-led review and follow-up, including medication review, interaction assessment, symptom triage, patient counseling, and ongoing monitoring during outpatient care.

Future Directions and Scalability

As the landscape of systemic anticancer therapy continues to evolve, the principles outlined in this review are likely to remain relevant across emerging treatment modalities. Antibody-drug conjugates are increasingly incorporated into earlier lines of therapy and combination regimens, often introducing gastrointestinal toxicity profiles that reflect both targeted and cytotoxic components and require structured outpatient monitoring. Similarly, the development of oral bispecific antibodies and next-generation small molecules may further increase reliance on prolonged outpatient administration, amplifying the importance of early toxicity recognition, pharmacokinetic awareness, and timely escalation. Combination regimens involving targeted therapies and immune checkpoint inhibitors introduce additional complexity, heightening the risk of misattribution when gastrointestinal symptoms arise. In these settings, a structured, pharmacology-informed approach that integrates mechanism, exposure modifiers, severity assessment, and escalation thresholds may be particularly valuable for maintaining treatment continuity while safeguarding patient safety. Future research may further address gaps in toxicity prediction, pharmacogenomic influences, and the development of prospective toxicity management models to support more individualized outpatient care.

Limitations

This review is narrative in design and did not include formal quantitative synthesis or structured risk-of-bias assessment. Conclusions are therefore based on integration of clinical practice guidelines, randomized trials, observational studies, and mechanistic literature rather than pooled effect estimates. In addition, emerging domains such as microbiome-mediated susceptibility to targeted therapy-associated diarrhea remain exploratory and should not be translated into routine clinical practice without stronger prospective evidence. Potential limitations also include literature selection bias inherent to narrative synthesis and reliance on PubMed/MEDLINE as the primary database, which may not capture all relevant sources. In addition, the maturity and depth of available evidence vary across targeted therapy classes, which may influence the strength of conclusions drawn for specific agents.

## Conclusions

Gastrointestinal toxicities remain a frequent and clinically consequential challenge in patients receiving targeted cancer therapies, with diarrhea representing the most prominent adverse event affecting outpatient tolerability. A pharmacology-informed approach that integrates class-specific toxicity patterns, drug-specific prophylaxis in high-risk settings, and management of pharmacokinetic interaction risks may improve symptom control and support treatment continuity. Pharmacists play a central role in implementing these strategies through structured outpatient triage, medication review, and escalation pathways that distinguish routine toxicity from high-risk events requiring urgent evaluation. In this context, the MESE framework supports practical clinical workflows by linking mechanism-based assessment and exposure review with severity-guided intervention and escalation decisions.
